# Armless hairpin-like tRNAs in *Romanomermis culicivorax*: Evolutionary adaptation of a mitochondrial elongation factor EF-Tu

**DOI:** 10.1016/j.jbc.2025.110294

**Published:** 2025-05-24

**Authors:** Dorian Bernier, Nadine Grafl, Josefine Gnauck, Heike Betat, Sebastian Dengler, Ivan Huc, Mario Mörl

**Affiliations:** 1 Institute for Biochemistry, Leipzig University, Leipzig, Germany; 2 Department of Pharmacy, Ludwig-Maximilians-Universität, Munich, Germany

**Keywords:** elongation factor EF-Tu1, armless tRNAs, compensatory co-evolution, adaptation, mitochondria

## Abstract

tRNAs are central players in translation, delivering cognate amino acids to the ribosome. To fulfill this essential function, secondary and tertiary structures of tRNAs are highly conserved. In metazoan, however, several mitochondrial tRNAs show strong structural deviations and lack D- or T-arms. As these elements are important for the interaction with tRNA-binding proteins, these proteins are adapted to recognize such unusual targets. A prominent example is mitochondrial EF-Tu, delivering aminoacylated tRNAs to the ribosome. In nematode mitochondria, two variants of mt-EF-Tu exist. While mt-EF-Tu2 is specific for D-armless mt-tRNA^Ser^, mt-EF-Tu1 recognizes the remaining 20 tRNAs. The most bizarre mt-tRNAs are found in *Romanomermis culicivorax*, where hairpin-like structures were described lacking both D- and T-arm. To ensure functional translation with such extremely reduced tRNAs, the corresponding mt-EF-Tu1 must have undergone a further adaptation. In a comparative analysis, the tRNA-binding behavior of recombinant mitochondrial EF-Tu1 versions from several nematodes was investigated. They all carry a C-terminal extension that is required for an efficient interaction with non-canonical tRNAs. Furthermore, in mt-EF-Tu1 from *R. culicivorax* and *Caenorhabditis elegans*, a basic residue in domain III was identified that represents an additional adaptation in the transition from canonical towards hairpin-like tRNA substrates. The results indicate that nematode mt-EF-Tu1 proteins are in principle able to interact with hairpin-like tRNAs, although such transcripts are only found in some of these species. Hence, concerning mt-EF-Tu, the evolutionary stage is set for a further truncation of mitochondrial tRNAs towards armless structures.

As adapters delivering amino acids to the ribosome, tRNAs are crucial elements in translation. In all domains of life, these transcripts form a highly conserved secondary structure consisting of an acceptor stem and an anticodon arm separated by a D- and T-arm. This cloverleaf-like structure further folds into an L-shaped tertiary form that is important for tRNA function as well as for its interaction with proteins involved in maturation, aminoacylation, and translation (1, 2, 3, 4).

In metazoan mitochondria, however, several tRNAs exhibit considerable deviations (Table 1). A mammalian mitochondrial tRNA^Ser^ that lacks the entire D-arm was described by Brujn *et al.* (9). Later, Wolstenholme discovered a set of T-armless tRNAs in the nematodes *Ascaris suum* and *Caenorhabditis elegans* (10). Sequencing of mitochondrial genomes from nematodes (5) and arthropods predicted the existence of even further truncated tRNA structures, where D- and T-arm are replaced by short single-stranded connector elements (5, 5, 11, 12, 13, 14, 15, 16). The mitochondrial genome of the nematode *Romanomermis culicivorax* encodes for a complete set of such predicted non-canonical tRNAs (Fig. S1), and several of the completely armless tRNAs were proven to be expressed *in vivo* and were successfully processed by a co-evolved tRNA nucleotidyltransferase (17, 18). Furthermore, the corresponding *in vitro* transcripts fold into a stable hairpin-like secondary structure and form a boomerang-like shape instead of the conventional L-form (19).

**Table 1:** Mitochondrial tRNA structures found or predicted in the investigated metazoan species

				
*R. culicivorax* (5)	0	11	2	9
*T. spiralis* (6)	6	14	2	0
*C. elegans* (7)	0	20	2	0
*B. taurus* (8)	21	0	1	0

References for the described mitochondrial tRNA shapes.

Due to these structural deviations, non-canonical tRNAs represent a challenge for processing and tRNA-binding proteins, resulting in compensating adaptations. The CCA-adding enzymes of several nematodes carry several basic residues in an element of the catalytic core as well as a C-terminal extension that both contribute to an increased binding affinity to structurally deviating tRNAs, while corresponding enzymes dealing exclusively with canonical tRNA structures lack these residues and do not accept armless tRNAs for CCA-addition (17, 20). A further example of a tRNA-interacting protein is EF-Tu, a translational elongation factor in bacteria and mitochondria, binding aminoacylated tRNAs (aa-tRNAs) and delivering them to the ribosome. EF-Tu is composed of three distinct domains that are separated by linker elements, enabling a certain flexibility for conformational changes. While every domain has a specific function, they work cooperatively to form a complex with aa-tRNAs. Domain I carries a guanosine nucleotide-binding pocket, the interface between domains I and II forms the amino acid binding site, and all three domains contribute to a tRNA-binding cleft (21). Conventional EF-Tus recognize the amino acid-carrying acceptor stem as well as the T-arm of a tRNA (22, 23). Hence, to interact with mitochondrial tRNAs lacking this arm, the mt-EF-Tu must have undergone a specific adaptation in its binding mode.

In contrast to most bacteria and mammals, nematodes (and some insects like *Drosophila melanogaster*) carry two distinct mitochondrial EF-Tu versions, mt-EF-Tu1 and mt-EF-Tu2, that differ in their binding properties. mt-EF-Tu2 specifically interacts with D-armless mt-tRNA^Ser^, where it recognizes the tRNA region lacking the D-arm as well as the seryl moiety (24, 25, 26, 27). In contrast, mt-EF-Tu1 recognizes all other aminoacyl-tRNAs in the pool. Interestingly, several nematode mt-EF-Tu1 proteins differ in their binding behavior. While mt-EF-Tu1 from *C. elegans* (*Cel* mt-EF-Tu1) is highly specific for T-armless tRNAs (28), the corresponding ET-Tu1 from *Trichinella britovi* (*Tbr* mt-EF-Tu1) recognizes canonical as well as D- and T-armless tRNAs (24). Compared to their conventional counterparts (from mammals or bacteria), the nematode mt-EF-Tu1 and mt-EF-Tu2 carry C-terminal extensions of different length that are essential for binding to their tRNA substrates as a deletion of these regions abolishes such interactions (29, 30).

While the C-terminal extension of mt-EF-Tu1 from *C. elegans* seems to interact with the D-arm of T-armless tRNAs (29), such a binding mode is not possible with hairpin-like tRNAs lacking both T- and D-arm as they are found in *R. culicivorax*. Hence, we investigated the binding behavior of the corresponding recombinant mt-EF-Tu1 (*Rcu* mt-EF-Tu1) and compared that to previously studied elongation factors. The results show that *Rcu* mt-EF-Tu1 but also *Tbr* mt-EF-Tu1 and *Cel* mt-EF-Tu1 are able to interact with armless tRNAs, although such transcripts do not exist in *C. elegans* or *T. britovi*.

In *Rcu* mt-EF-Tu1, two elements are responsible for this specific binding. Besides a C-terminal extension of 53 amino acid positions, a basic residue in domain III is essential for tRNA recognition. Surprisingly, the canonical EF-Tu proteins carry an acidic residue at this position that contributes to the interaction with conventional tRNAs. The change from this acidic to a basic side chain represents a further adaptation in the amazing co-evolution of hairpin-like tRNAs and their corresponding mt-EF-Tu1 in *R. culicivorax*.

## Results

### *R. culicivorax* carries two versions of mitochondrial elongation factor EF-Tu

**Figure 1:**
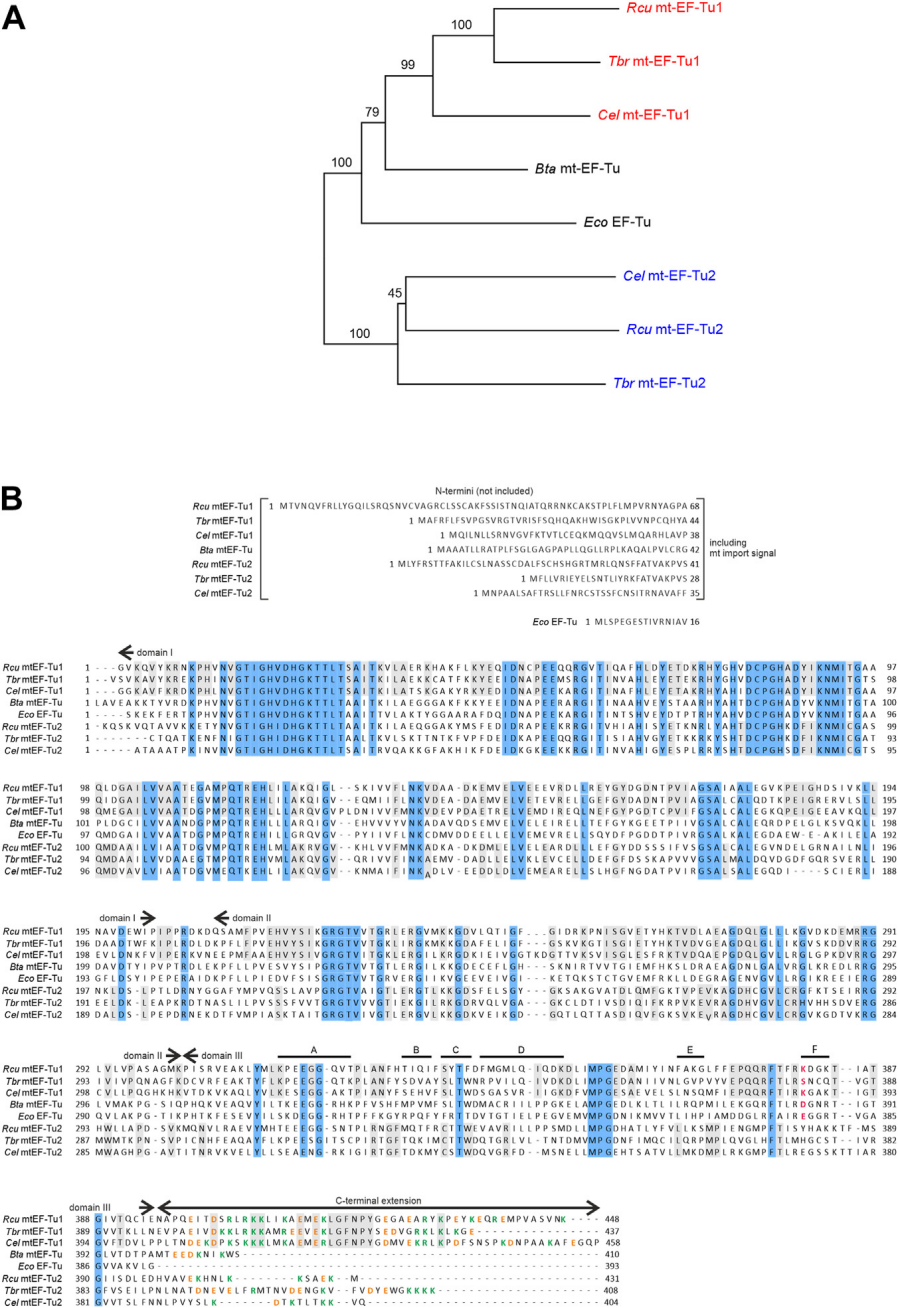
**EF-Tu proteins investigated in this study.***A*, phylogenetic tree of mitochondrial EF-Tu proteins from nematodes (*Rcu*, *R. culicivorax*; *Tbr*, *T. britovi*; *Cel*, *C. elegans*), a mammalian representative (bovine mt-EF-Tu from *B. taurus; Bta*) and a bacterial version (*Eco*, *E. coli*) as an outgroup. The nematode mt-EF-Tu1 and mt-EF-Tu2 versions cluster together, indicating that *R. culicivorax* also carries two EF-Tu versions that are closely related to those of *C. elegans* and *T. britovi*. *B*, sequence alignment of nematode mt-EF-Tu1 and mt-EF-Tu2 versions with *Bta* mt-EF-Tu and *Eco* EF-Tu. N-termini including the mitochondrial import signal are shown separately and were not included in the cloning. Identical regions in all proteins are indicated in cyan and are predominantly found in domains I and II, while domain III shows more deviations. Conserved positions in mt-EF-Tu1 and mt-EF-Tu2, respectively, are indicated in *grey*. The exchanged regions A to F of domain III are indicated. C-terminal extensions as well as a tRNA-interacting position (*red*) in domain III are indicated in mt-EF-Tu and mt-EF-Tu1 but are absent/not identified in mt-EF-Tu2 proteins. Basic and acidic residues in the C-terminal extension are labeled in *green* and *orange*, respectively.

Using highly conserved regions between EF-Tus from bacteria and mitochondria, two distinct mt-EF-Tu sequences were identified in *R. culicivorax* transcriptome data (Fig. 1) (31). As described for other nematodes (6, 24, 25), these two proteins were closely related to mt-EF-Tu1 and mt-EF-Tu2, clustering in the same branches of a phylogenetic tree (Fig. 1*A*). Thus, a sequence alignment of *R. culicivorax* mt-EF-Tu versions with the corresponding nematode proteins indicates a high sequence similarity in the N-terminally located domains I and II (Fig. 1*B*). As *R. culicivorax* carries two mitochondrially encoded tRNAs for serine lacking the D-arm, the situation is highly similar to that of other nematodes (5, 24, 25). Accordingly, the recombinant *Rcu* mt-EF-Tu2 proved to be serine-specific and was not further investigated in this study (Fig. S2).

Compared to mt-EF-Tu1 from *C. elegans* (*Cel* mt-EF-Tu1) and *T. britovi* (*Tbr* mt-EF-Tu1), *Rcu* mt-EF-Tu1 exhibits a sequence identity of 56% and 66%, respectively, while it shares 53% identity with *Bos taurus* mt-EF-Tu (*Bta* mt-EF-Tu) and 47% with *Escherichia coli* EF-Tu (*Eco* EF-Tu). Similar to the corresponding nematode proteins, *Rcu* mt-EF-Tu1 carries a C-terminal extension, composed of 53 residues. Hence, it is slightly shorter than the extension of *Cel* mt-EF-Tu1 (57 residues) but longer than that of *Tbr* mt-EF-Tu1 (41 residues). Although the sequence is not highly conserved between the individual nematode proteins (41% and 51% identity with *Cel* mt-EF-Tu1 and *Tbr* mt-EF-Tu1, respectively), these extensions exhibit an enrichment of basic as well as acidic residues (Fig. 1*B*).

### Mt-EF-Tu1 from *R. culicivorax*, *C. elegans* and *T. britovi* exhibit a high affinity toward armless tRNAs

To investigate the interaction of *Rcu* mt-EF-Tu1 and armless tRNAs, an increasing amount of recombinant purified protein was incubated with internally labeled and aminoacylated transcripts. For aminoacylation of the transcribed tRNAs, the eFx flexizyme approach was used (32). This artificial ribozyme showed the highest aminoacylation rate with activated phenylalanine cyanomethyl ester (Phe-CME) (33). Hence, regardless of identity, all tRNA transcripts were charged uniformly with phenylalanine. The combination of phenylalanine with a non-cognate tRNA can in principle skew the binding affinity of EF-Tu proteins, as tRNA–amino acid pairs exhibit a thermodynamic compensation, where a weakly bound amino acid is compensated by a strong binding of its cognate tRNA, and *vice versa* (34). Nevertheless, mischarging the individual tRNAs with phenylalanine in this case has the advantage that it allows to identify the impact of each tRNA structure on the binding behavior of the tested EF-Tu versions without a bias resulting from different amino acids bound to the tRNAs (35).

**Figure 2:**
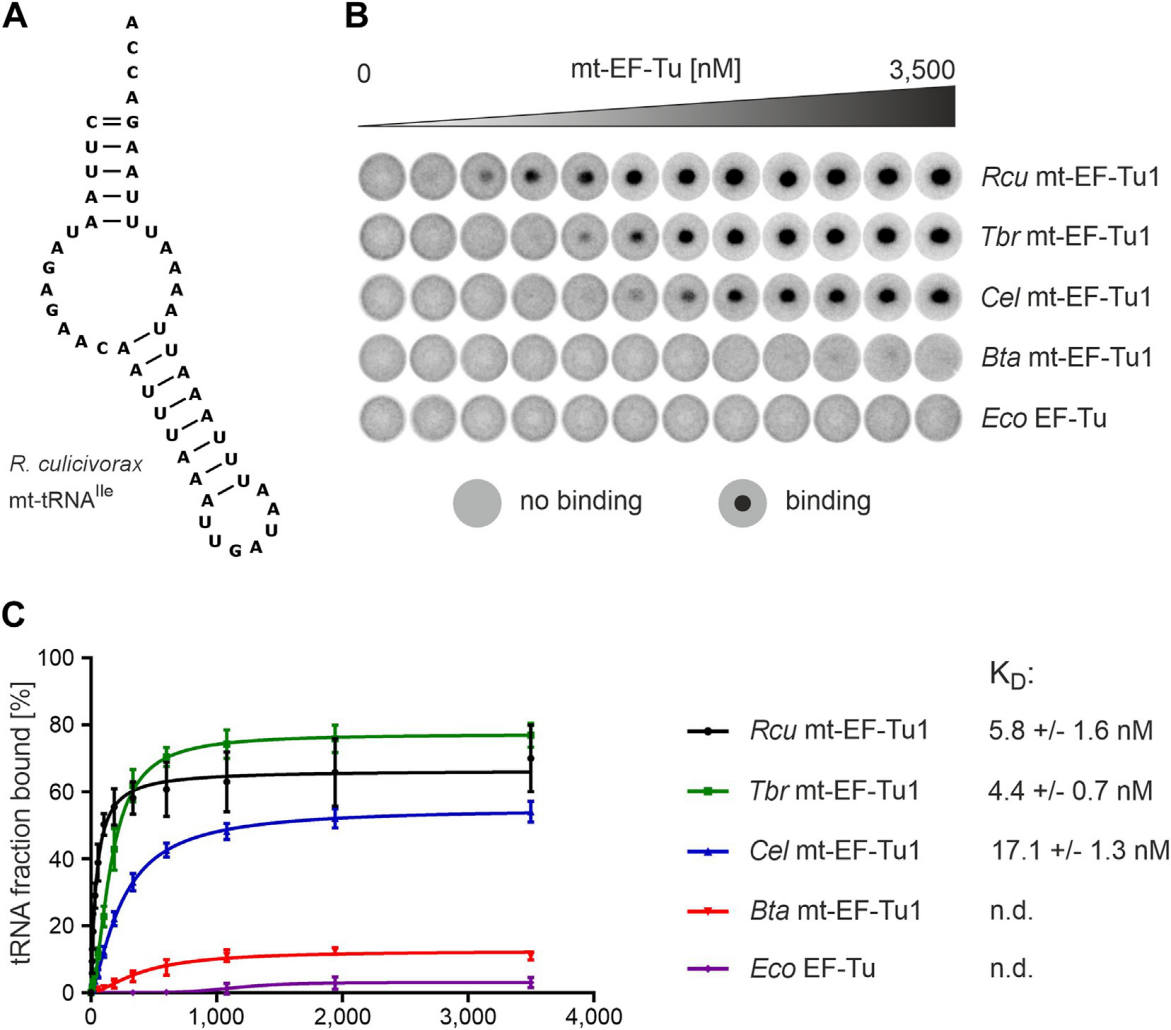
**Interaction of EF-Tu proteins with armless hairpin-like aminoacylated mt-tRNA^Ile^ from *R. culicivorax*.***A*, secondary structure of mt-tRNA^Ile^ as determined by Jühling *et al.* (19). D- and T-arms are not present. Instead, single-stranded connector elements link acceptor- and anticodon arms. *B*, DRaCALA test with ^32^P-labeled tRNA^Ile^ charged with phenylalanine demonstrates that only the nematode mt-EF-Tu1 proteins specifically interact with the armless tRNA, while the corresponding proteins from *B. taurus* mitochondria and *E. coli* show no interaction at all. *C*, binding curves resulting from DRaCALA experiments. The nematode mt-EF-Tu1 proteins show a high binding affinity to the armless aa-tRNA, ranging from 5.8 nM for *Rcu* mt-EF-Tu1 to 4.4 and 17.1 nM (for *Tbr* and *Cel* mt-EF-Tu1, respectively). In contrast, no binding constants could be determined for *Bta* mt-EF-Tu and *Eco* EF-Tu (n.d.). Error bars represent standard deviation (SD).

After formation of the ternary complex consisting of EF-Tu, GTP, and aa-tRNA, dissociation constants were determined. To this end, the DRaCALA approach was used as a highly robust and reproducible method (36, 37), as conventional assays for EF-Tu analysis like RNase- or deacylation-protection (28, 35, 38) rely on labeled amino acids and were therefore not suitable in our approach with labeled tRNA. Using the aminoacylated mt-tRNA for isoleucine from *R. culicivorax* (Fig. 2*A*) (19), the nematode proteins *Rcu* mt-EF-Tu1, *Tbr* mt-EF-Tu1 and *Cel* mt-EF-Tu1 exhibit efficient binding to the armless aa-tRNA (Fig. 2*B*). In contrast to this, proteins from organisms with conventional tRNAs (*Bta* mt-EF-Tu, *Eco* EF-Tu) show no binding to this non-canonical substrate. For *Rcu* mt-EF-Tu1, a dissociation constant K_D_ of 5.8 nM was determined, while the proteins from *T. britovi* and *C. elegans* have similar and slightly increased K_D_ values of 4.4 nM and 17.1 nM, respectively, indicating a comparable binding efficiency. These interactions are specific for mt-EF-Tu1:GTP, as mt-EF-Tu1 did not form a ternary complex with GDP or non-aminoacylated tRNA (Fig. S3).

### *Rcu* mt-EF-Tu1 prefers hairpin-like and T-arm-lacking tRNA structures

**Figure 3:**
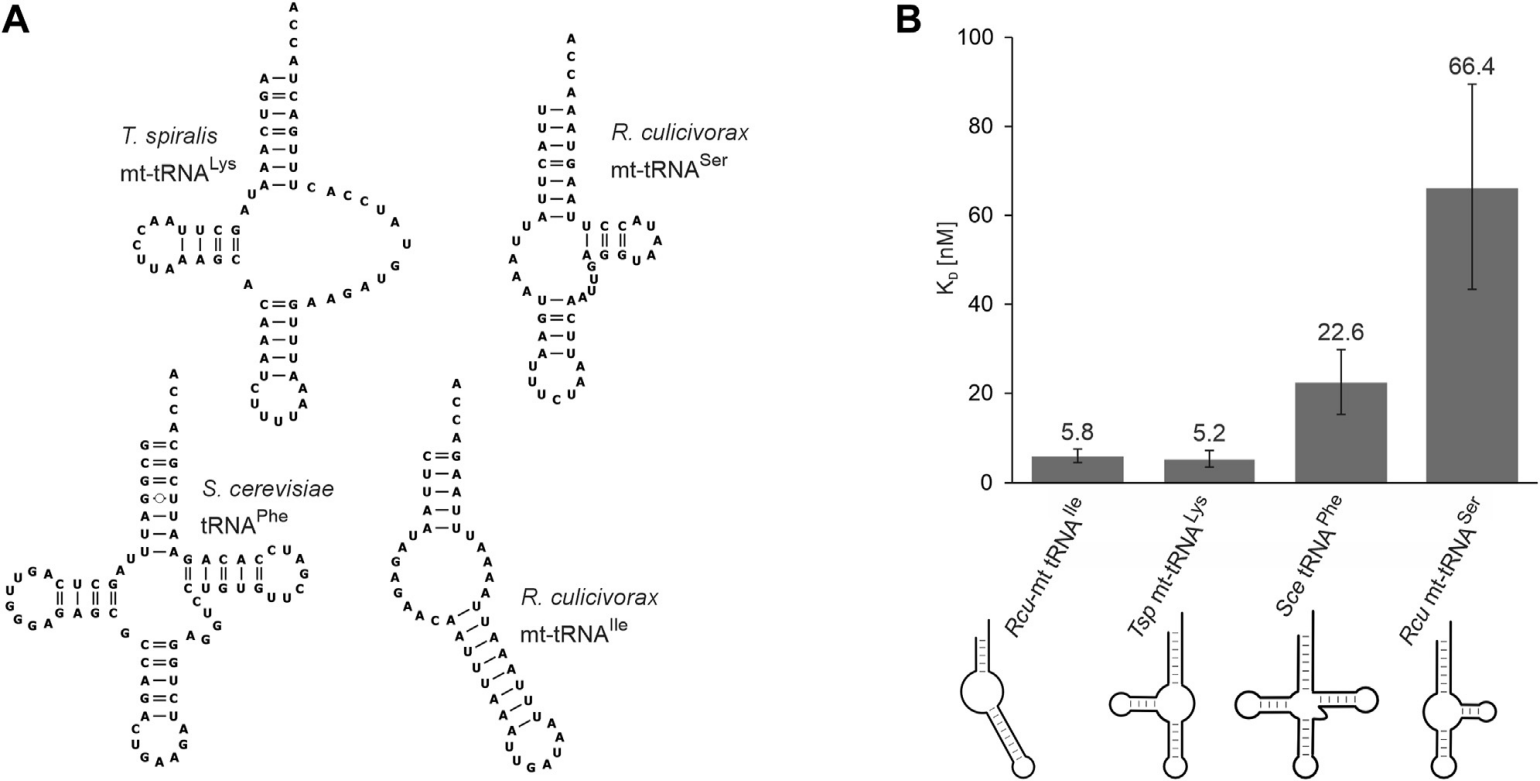
**Interaction of *Rcu* mt-EFTu1 with different aa-tRNA structures.***A*, mt-tRNAs lacking D-, T- or both arms from *R. culicivorax* and *T. spiralis*. A tRNA with canonical structure is represented by *Saccharomyces cerevisiae* tRNA^Phe^. *B*, binding constants of *Rcu* mt-EF-Tu1 for different aa-tRNA substrates. The protein exhibits the highest affinities for tRNA lacking the T- or both arms, while the canonical tRNA^Phe^ from yeast is somewhat less efficiently bound. In contrast, the D-arm lacking mt-tRNA^Ser^ shows a considerable drop in affinity, corroborating the fact that this tRNA is specifically bound by the serine-specific mt-EF-Tu2. Error bars represent standard deviation (SD).

The individual nematode mt-EF-Tu1 proteins exhibit certain differences in binding to their natural substrate tRNAs with deviating structures. In *C. elegans*, all mt-tRNA substrates for mt-EF-Tu1 lack the T-arm (28). In the case of *Tbr* mt-EF-Tu1, the substrate specificity is more relaxed, as it recognizes truncated as well as canonical tRNAs (24). To investigate the substrate specificity of *Rcu* mt-EF-Tu1, several representative types of aminoacylated tRNA structures were offered to the recombinant protein (Fig. 3*A*). As representatives for armless hairpin-like and D-armless tRNAs, mitochondrial tRNA^Ile^ and tRNA^Ser^(UCU) from *R. culicivorax* were chosen. The canonical cloverleaf tRNA structure was represented by yeast tRNA^Phe^, a transcript that is frequently used as a model tRNA with well-characterized secondary and tertiary structure (39). In *R. culicivorax* mitochondria, T-armless tRNAs are currently only predicted but not yet identified at the RNA level. Hence, mitochondrial T-arm-lacking tRNA^Lys^ from *Trichinella spiralis* was selected, as this transcript was already tested as a substrate for *Tbr* mt-EF-Tu1 (24).

The DRaCALA analysis showed that *Rcu* mt-EF-Tu1 has a very high affinity for the T-armless and completely armless tRNAs, with a K_D_ of 5.2 and 5.8 nM, respectively (Fig. 3*B*). For the canonical yeast tRNA^Phe^, the dissociation constant increased 4-fold to 22.6 nM, and it showed a further 12-fold increase (66.4 nM) for the D-arm lacking tRNA^Ser^(UCU). These results correlate with the tRNA situation in *R. culicivorax* mitochondria, where no canonical tRNAs are found, and the D-armless tRNA^Ser^ is bound by the serine-specific mt-EF-Tu2 (Fig. S2). Hence, *Rcu* mt-EF-Tu1 is specifically adapted to armless and T-armless tRNAs and exhibits a very high binding affinity to these heavily truncated transcripts.

### The C-terminal extension of *Rcu* mt-EF-Tu1 is essential for binding to armless tRNAs but not to canonical tRNAs

A typical feature in nematode mt-EF-Tu1 proteins is a C-terminal extension, which is discussed to represent an additional binding interface between the protein and a tRNA that lacks the typical elbow region of canonical tRNAs (24, 29). As a compensation for the lacking interaction with the missing T-arm, this extension recognizes the D-arm of such tRNA substrates (29). Since nine mitochondrial tRNAs of *R. culicivorax* have a hairpin-like structure with connector elements instead of T- and D-arm, we investigated whether the corresponding C-terminal extension in *Rcu* mt-EF-Tu1 contributes to the binding to these further truncated tRNAs.

**Figure 4:**
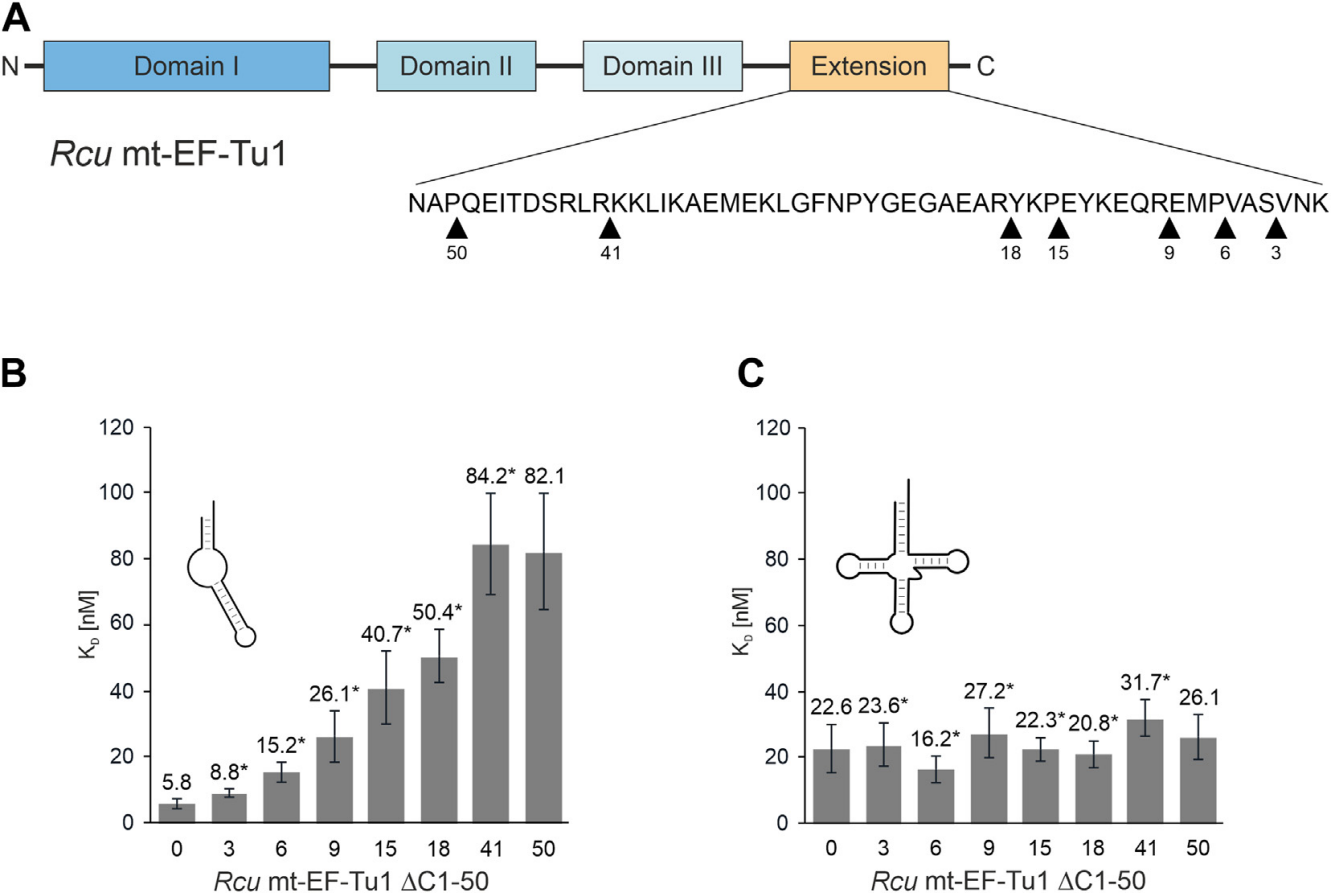
**The C-terminal extension of *Rcu* mt-EF-Tu1 significantly contributes to the binding of armless tRNA but is not involved in the interaction with canonical tRNA structures.***A*, domain organization of *Rcu* mt-EF-Tu1. Domains I to III are indicated in *blue* colors, the C-terminal extension is indicated in *orange*. *Blackarrow* heads indicate the C-termini of the individual deletion variants, where stop codons were introduced into the coding region. The numbers (starting from the original C-terminus) indicate how many amino acid residues have been removed. *B*, binding affinities of individual C-terminal deletion variants. A gradual increase in the determined binding constants indicates that the whole extension contributes to the interaction with armless aa-tRNA. *C*, in contrast, gradual deletions of the C-terminal extension do not affect binding to a canonical aa-tRNA, indicating that the extension represents a specific adaptation to armless tRNA structures. Error bars represent standard deviation (SD) An *asterisk* indicated apparent K_D_ values, where the active fraction of the wt protein was used for calculation.

To this end, variants of *Rcu* mt-EF-Tu1 with increasing deletions of the C-terminal extension were generated (Fig. 4*A*). The positions where stop codons were introduced into the cloned open reading frame were arbitrarily chosen across the whole extension, resulting in deletion variants *Rcu* mt-EF-Tu1 ΔC1 to ΔC50. For reasons of clarity, this nomenclature, according to Lusetti *et al.,* was chosen (40). A stepwise increase in the C-terminal deletion led to a corresponding gradual loss of binding affinity toward aminoacylated armless mt-tRNA^Ile^ (Fig. 4*B*). While deleting the three terminal amino acid residues (ΔC3) leads to a moderate 1.5-fold increase of apparent K_D_ values, further deletions result in a stepwise reduction of affinity to up to 14-fold for *Rcu* mt-EF-Tu1 ΔC50. In contrast to ΔC18 with an 8.7-fold loss of affinity, the deletion of the remaining 32 positions (ΔC50) only leads to a further 1.6-fold loss. This indicates that the very terminal 18 positions make a greater contribution to armless tRNA binding than the residual, internal part of the extension (32 residues).

In contrast to this gradual reduction in binding to the armless tRNA substrate, none of the C-terminal deletions affected the affinity for the canonical tRNA structure (Fig. 4*C*). This is a strong indication that the C-terminal extension in *Rcu* mt-EF-Tu1 represents a specific adaptation to the non-canonical tRNA, while the conventional tRNA is probably bound *via* the tRNA cleft formed by domains I to III as described by Nissen *et al.* (21).

### A basic residue in domain III of *Rcu* mt-EF-Tu1 contributes to tRNA binding

While the C-terminal extension of *Rcu* mt-EF-Tu1 is important for the interaction with armless tRNAs, its deletion does not result in a complete loss of binding to these hairpin-like tRNAs. Hence, additional regions in the protein contribute to this interaction. As bacterial and mitochondrial EF-Tu proteins fold into three domains I to III with very similar 3D architecture, this additional adaptation must be represented by minor changes in the sequence. Here, domain III shows the least conservation of all domains (Fig. 1*A*). In addition, this domain is involved in recognizing the elbow region of canonical tRNAs (21), an element that is not present in armless tRNAs (18). Hence, the tRNA binding capacity of domain III of *Rcu* mt-EF-Tu1 was further investigated.

**Figure 5:**
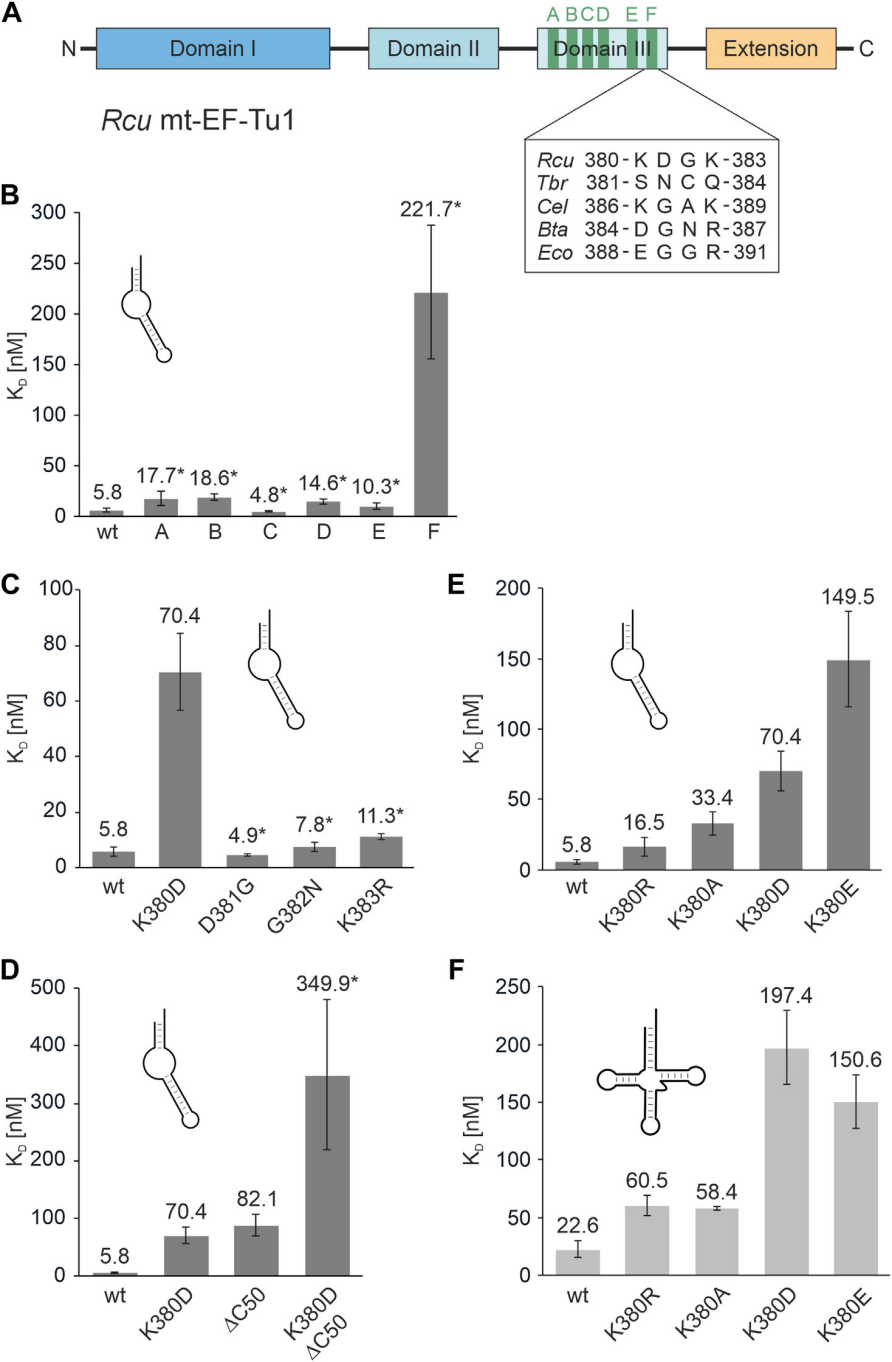
**A basic residue in region F of domain III is involved in mt-EF-Tu1 binding to armless aa-tRNA.***A*, regions A to F of *Rcu* mt-EF-Tu1 were individually replaced by the corresponding regions of *Bta* mt-EF-Tu (*green*). Sequence composition of region F in the individual EF-Tu versions is indicated. The determined K_D_ values of this figure indicate that the first position of this region (K380) specifically contributes to the interaction with armless or canonical aa-tRNAs, respectively. *B*, in *Rcu* mt-EF-Tu1, only the replacement of region F leads to a dramatic increase in the binding constant, demonstrating that this region is involved in binding of armless aa-tRNA. *C*, amino acid replacements in region F of *Rcu* mt-EF-Tu1 by the corresponding residues of *Bta* mt-EF-Tu. Only the replacement at position 380 interferes with binding, resulting in a strong increase in K_D_, while amino acid exchanges at positions 381 to 383 are tolerated. *D*, Both region F and the C-terminal extension are involved in armless aa-tRNA interaction. If both elements are simultaneously replaced/deleted (K380D/ΔC50), the affinity drops dramatically to 349.9 nM, indicating a cumulative effect. *E*, in *Rcu* mt-EF-Tu1, the basic character of position 380 is important for armless aa-tRNA interaction. While replacing lysine by arginine only shows a small effect on K_D_, a neutral alanine residue at this position leads to a six-fold loss in affinity. If acidic residues aspartate or glutamate are introduced, as they are found in *Bta* mt-EF-Tu1 and *Eco* EF-Tu, binding to armless tRNA is severely affected, resulting in a 12- to 28-fold increase in K_D_. *F*, K380D or K380E do not enhance binding of *Rcu* mt-EF-Tu1 to canonical aa-tRNA. Instead, these replacements show a similar detrimental effect as observed for armless tRNA binding. This result indicates that in *Rcu* mt-EF-Tu1, the mode of tRNA interaction differs dramatically from that in *Bta* mt-EF-Tu or *Eco* EF-Tu. *Asterisks* indicate apparent K_D_ values. Error bars represent standard deviation (SD).

To this end, individual regions of domain III were replaced by the homologous parts of mt-EF-Tu from *B. taurus*, the most closely related EF-Tu studied so far that does not show an adaptation to armless tRNAs (Fig. 2). As no structural data on the ternary complex of a mitochondrial EF-Tu are available, the structure of the corresponding complex from *E. coli* EF-Tu and yeast tRNA^Phe^ was used to identify regions with low conservation but in close contact with the bound tRNA (pdb entry 1OB2). In total, six regions A to F from *Bta* mt-EF-Tu were introduced into *Rcu* mt-EF-Tu1 to replace the original sequences (Fig. 1*B*; Fig. 5*A*). All chimeric proteins were active and five of them (carrying regions A to E) exhibited an unimpaired high affinity toward the aminoacylated armless tRNA substrate, resulting in an apparent K_D_ similar to that of the wild-type protein (Fig. 5*B*). Only region F led to a dramatic loss of affinity, with an apparent dissociation constant of 221.7 nM, corresponding to a 38-fold increase relative to the wt situation (K_D_ = 5.8 nM).

In the bacterial EF-Tu structure, region F corresponds to a turn in an antiparallel beta-sheet directly pointing toward the T-stem of the bound tRNA (21) (Fig. S4). In *Rcu* mt-EF-Tu1, this region consists of four amino acid residues KDGK (Fig. 5*A*). To further investigate its contribution to tRNA binding, the individual positions were replaced by the corresponding residues D, G, N, and R from *Bta* mt-EF-Tu. Only the replacement K380D led to an impairment of tRNA binding, resulting in a K_D_ of 70.4 nM (Fig. 5*C*). Interestingly, this replacement also affected the interaction with the canonically structured tRNA (Fig. 5*F*), while the deletion of the C-terminal extension only affected armless tRNA recognition (Fig. 4). A combination of both C-terminal deletion and K380D led to a cumulative reduction in affinity with an apparent dissociation constant of 349.9 nM (Fig. 5*D*).

To investigate whether the basic character of K380 is an important feature contributing to tRNA recognition, this position was replaced by amino acid side chains with positive (arginine), negative (aspartate, glutamate), or neutral charge (alanine) (Fig. 5*E*). Replacing lysine by the also positively charged arginine had only a moderate effect on armless tRNA binding, with a 2.8-fold increase in K_D_ (16.5 nM), while the neutral alanine led to a 5.8-fold loss of affinity (K_D_ = 33.4 nM). The negatively charged aspartate and glutamate residues, however, exhibited the strongest effect, with a 12- and 26-fold increase in K_D_, respectively (70.4 and 149.5 nM). Similar effects were observed when a canonical tRNA was offered as a substrate (Fig. 5*F*).

### In various EF-Tu proteins, position 380 contributes to tRNA recognition in different ways

**Figure 6:**
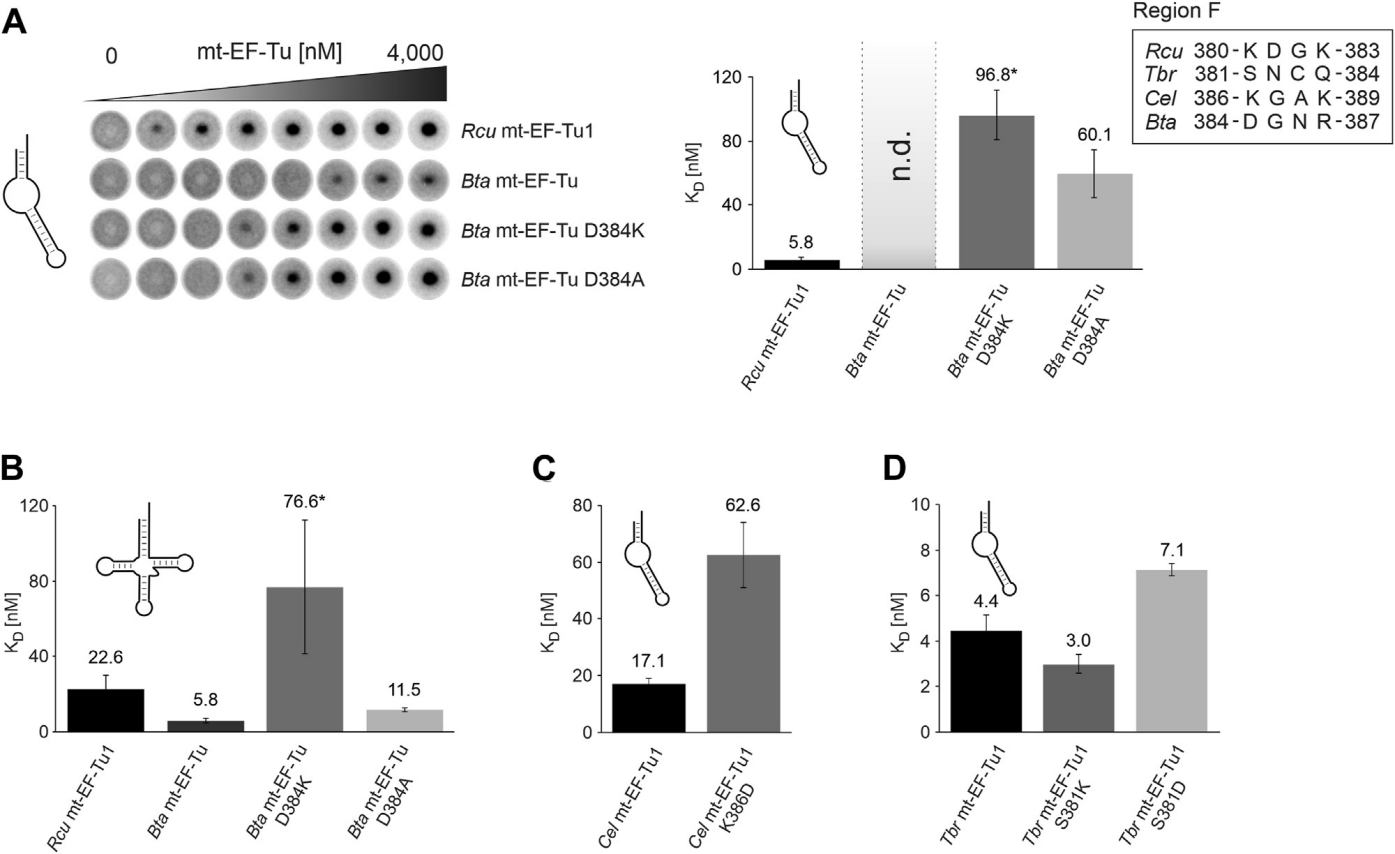
**Position one in region F contributes differently to tRNA binding.***A*, when the acidic residue D384 is replaced by lysine or alanine, the resulting *Bta* mt-EF-Tu protein can bind to an armless aa-tRNA at moderate affinity with binding constants of 96.8 and 60.1 nM, respectively. The wt protein, in contrast, shows no measurable binding (n.d.). *B*, while the introduction of a neutral alanine residue (D384A) in *Bta* mt-EF-Tu only leads to a two-fold increase in K_D_ for a canonical aa-tRNA, the basic replacement D384K strongly impairs binding and leads to a 13-fold loss of affinity. *C*, in *Cel* mt-EF-Tu1, a replacement of the corresponding lysine position by aspartate (K386D) reduces the binding affinity for armless aa-tRNA about three-fold, indicating a contribution of K386 to this substrate interaction similar to that observed in *Rcu* mt-EF-Tu1. *D*, in contrast, the corresponding position in *Tbr* mt-EF-Tu1 is less involved in armless aa-tRNA binding. Replacing the original serine position by lysine (S381K) does not affect tRNA interaction, and the introduction of an acidic aspartate (S381D) shows only a moderate 1.6-fold increase in K_D_. *Asterisks* indicate apparent K_D_ values. Error bars represent standard deviation (SD).

The opposing effects of positively and negatively charged residues at position 380 in *Rcu* mt-EF-Tu1 could explain the inability of *Eco* EF-Tu and *Bta* mt-EF-Tu to form a complex with an armless tRNA (Fig. 2), as both proteins not only lack a C-terminal extension but also carry acidic residues at the corresponding positions (E388 in *Eco* EF-Tu and D384 in *Bta* mt-EF-Tu; Figs. 1*B* and 5*A*). To test this hypothesis, the aspartic acid residue in *Bta* mt-EF-Tu was replaced by lysine, resulting in *Bta* mt-EF-Tu D384K. While the wt protein shows no interaction with the armless tRNA, the variant D384K binds this tRNA with an apparent K_D_ of 96.8 nM (Fig. 6*A*). With a canonical tRNA, however, this replacement results in a 13-fold weaker binding (K_D_ = 76.6 *versus* 5.8 nM), indicating the importance of this acidic residue for an interaction with a cloverleaf-like tRNA (Fig. 6*B*). Interestingly, when D384 was replaced by the neutral amino acid alanine, the resulting protein showed an increased binding to the armless tRNA and only a slightly decreased interaction with the conventional tRNA substrate with K_D_ values of 60.1 and 11.5 nM, respectively (Fig. 6, *A* and *B*). While these dissociation constants still indicate a considerable binding behavior of the mutant *Bta* EF-Tu, armless tRNA binding of wt *Rcu* mt-EF-Tu1 is substantially stronger, highlighting the importance of the C-terminal extension in the recognition of armless tRNAs. Yet, in the context of the bovine mt-EF-Tu, the introduction of this extension (*Bta* mt-EF-Tu/*Rcu* C50) did not result in efficient binding of the armless tRNA substrate (Fig. S5). Overall, our results show that the analyzed position in domain III (position 380 in *Rcu* mt-EF-Tu1 and 384 in *Bta* mt-EF-Tu) contributes to tRNA binding in both proteins. In the *R. culicivorax* protein, the positively charged K380 enhances the binding to armless as well as conventional tRNAs. In the *B. taurus* protein, however, the negatively charged D384 only supports binding to the conventional tRNA, while it strongly interferes with the recognition of the armless tRNA substrate.

Surprisingly, while the other nematode proteins *Cel* mt-EF-Tu1 and *Tbr* mt-EF-Tu1 that also recognize armless tRNAs all carry similar C-terminal extensions, the nature of the position corresponding to D380 is not conserved. Like in *R. culicivorax*, the *C. elegans* protein carries a lysine residue K386, and a change to aspartic acid (K386D) led to a 3.6-fold increase of the K_D_ for the armless tRNA from 17.1 nM to 62.6 nM (Fig. 6*C*), indicating that K386 contributes to tRNA binding. In the *T. britovi* counterpart, however, a serine residue is found at position 381, and a replacement by lysine did not affect the K_D_ (3.0 nM for S381K *versus* 4.4 nM for the wt protein; Fig. 6*D*). When an aspartate residue was introduced (S381D), a slight 1.6-fold increase in K_D_ (7.1 nM) was observed (Fig. 6*D*).

Taken together, these results reveal that domain III - especially region F - represents an important part of mitochondrial EF-Tu proteins to interact with canonical as well as structurally reduced tRNAs. In *Rcu* and *Cel* mt-EF-Tu1, a lysine at the first position of this region is here of particular importance. In the bovine protein, an acidic residue supports selective binding of canonical tRNAs, while its replacement by the basic lysine enhances the interaction with armless tRNA. However, in contrast to the C-terminal extension, the involvement of domain III in this substrate specificity is less conserved in nematode mt-EF-Tu1 proteins.

## Discussion

Besides the CCA-adding enzyme of *R. culicivorax* (17), *Rcu* mt-EF-Tu1 is the second protein proven to specifically interact with armless mitochondrial tRNAs, providing further evidence that such hairpin-like tRNAs are functional components of the translational machinery in nematode mitochondria (18, 19). While the cloverleaf-like tRNA shape is considered a highly conserved feature (2, 41), there is an increasing number of examples describing tRNAs with considerable size reduction and deletions of D- or T-arm regions (Table 1) (42, 43). Especially, metazoan mitochondria are enriched in such bizarre tRNAs, and the first example to be identified was mammalian mt-tRNA^Ser^ lacking the D-arm (9). In nematodes and arachnids, this situation comes to an extreme, as most of the mitochondrial tRNAs lack the D- or the T-arm, and—in some cases—even both arms, resulting in hairpin-like tRNAs (7, 10, 11, 13, 14, 16, 18, 44). Accordingly, mitochondrial tRNA-interacting proteins like translation factor EF-Tu must have undergone a specific adaptation to recognize these structurally deviating tRNAs (45). In canonical tRNAs, where the D- and T-arm form the elbow region in the three-dimensional L-shape of the tRNA, domain III of this protein specifically interacts with the T-arm (21). However, in tRNAs lacking the T-arm, this interaction is not possible, and in nematodes like *C. elegans* and *T. britovi*, mt-EF-Tu1 carries a specific C-terminal extension to bind such tRNAs *via* D-arm interaction, compensating for the missing T-arm (6, 24, 45). In *R. culicivorax* mitochondria, however, further reduced tRNAs with connector regions instead of D- and T-arm were identified (18, 19). Hence, a binding strategy based on D-arm interaction is not possible in this case. Yet, our results indicate that the investigated mt-EF-Tu1 proteins of *C. elegans*, *T. britovi*, and especially *R. culicivorax*, are able to interact with such hairpin-like tRNAs. With dissociation constants of 5.8 nM (*Rcu* mt-EF-Tu1), 4.4 nM (*Tbr* mt-EF-Tu1) and 17.1 nM (*Cel* mt-EF-Tu1), these proteins exhibit binding affinities similar to that of EF-Tu proteins that recognize canonical tRNAs, like *Eco* EF-Tu, *Bta* mt-EF-Tu or the corresponding protein of *Thermus thermophilus* (38, 46, 47). While mitochondrial T-armless tRNAs in *C. elegans* require an m^1^A modification at position nine for proper folding and mt-EF-Tu1 interaction (48, 49), a corresponding modification is obviously no prerequisite in the case of the armless mt-tRNA^Ile^ from *R. culicivorax*. As shown in Figure 2, all tested nematode mt-EF-Tu1 proteins exhibit efficient binding to the unmodified *in vitro* transcribed version of this tRNA substrate.

The high binding affinity of *Rcu* mt-EF-Tu1 is the result of at least two protein regions. The first one is represented by a C-terminal extension of 53 amino acid residues. Comparable extensions with similar patterns of basic and acidic side chains are described for mt-EF-Tu1 proteins from *C. elegans* (57 residues) and *T. britovi* (41 residues), where the positively charged basic residues seem to interact with the negatively charged phosphate backbone of the tRNA (Fig. 1*B*) (24, 29). For *Cel* mt-EF-Tu1, experimental evidence suggests that this extension binds to the tRNA D-arm, a compensation for the lacking T-arm that is recognized by EF-Tu proteins binding to canonical tRNAs. Yet, as hairpin-like tRNAs do not carry this structural element, the extension must also bind to a separate, unidentified, tRNA part. The different binding behavior is further supported by the fact that a deletion of the 14 C-terminal residues of *Cel* mt-EF-Tu1 abolishes binding to T-armless tRNA (29), whereas a comparable deletion of 15 residues in *Rcu* mt-EF-Tu1 increases the dissociation constant only 8-fold to 40.7 nM, which is still an efficient interaction with an armless tRNA substrate. Furthermore, as the increase in the deletion correlates with a decrease in binding affinity (Fig. 4*B*), it seems that the whole C-terminal extension of *Rcu* mt-EF-Tu1 is involved in the recognition of armless tRNAs, while it does not contribute to the binding of canonical cloverleaf-like tRNAs (Fig. 4*C*). This is a strong indication that the extension represents a specific adaptation to non-canonical tRNA substrates, and especially to hairpin-like tRNAs. However, as the D-arm is lacking in these tRNAs, an interaction of the *Rcu* mt-EF-Tu1 extension as described for *Cel* mt-EF-Tu1 (29) is not possible. Yet, our data also show that *Cel* mt-EF-Tu1 and *Tbr* mt-EF-Tu1 interact with an armless tRNA substrate with similar affinities (Fig. 2, *B* and *C*). Hence, it seems that the C-terminal extension can also bind to tRNA regions other than the D-arm, and a certain flexibility of this protein part could lead to a close contact with the connector region of the boomerang-shaped armless tRNA.

Such protein extensions as binding interfaces for non-canonical tRNAs are also found in other proteins that interact with structurally deviating tRNAs. A recent study of the human mt-SerRS revealed that it carries additional N- and C-terminal domains that interact with the stacked acceptor and T-arm of mt-tRNA^Ser^ *via* shape and charge complementarity (50). Another example is SelB, the specific elongation factor for tRNA for selenocysteine. This tRNA is also structurally deviating, as it carries an extra arm instead of the variable loop and an extended acceptor stem (8–9 base pairs instead of 7), leading to an increased size of about 100 nts (51). Interestingly, while SelB has a similar domain architecture to EF-Tu, it also carries an additional C-terminal extension of domain III required for specific recognition of tRNA^Sec^ (52). Furthermore, an additional domain IV conveys binding of SelB to the SECIS element in selenoprotein mRNA to deliver tRNA^Sec^ to the site of selenocysteine incorporation during translation. Hence, it seems that acquiring such extensions represents a common compensatory strategy of proteins to enable an efficient interaction with their unusual tRNA (and mRNA) substrates.

A second region that coevolved with armless tRNAs and that shows a strong contribution to efficient binding of these substrates was identified in domain III of *Rcu* mt-EF-Tu1 (Fig. 1*B*). Nissen and coworkers identified that this domain interacts with the elbow region of canonical tRNAs, a region that is formed by D- and T-arm (21, 53). Again, as this structural part does not exist in tRNAs lacking one or both arms, domain III has undergone a specific adaptation. This was demonstrated by Ohtsuki *et al.* who showed that fusing the C-terminal extension of *Cel* mt-EF-Tu1 to *Bta* mt-EF-Tu did not result in an efficient recognition of T-armless tRNAs (28). Only when domain III was additionally replaced by the nematode version, the resulting chimeric protein was able to bind to a T-armless tRNA. Our results on *Bta* mt-EF-Tu fused to the C-terminal extension of *Rcu* mt-EF-Tu1 corroborate these findings (Fig. S5), and they are further supported by our mutagenic analysis of domain III, where K380 was identified to contribute to the binding of armless tRNAs. The positive charge of this position suggests an electrostatic interaction with the tRNA. This charge also contributes to the interaction with a canonical tRNA (Fig. 5*F*), while negative charges strongly reduce the affinity of *Rcu* mt-EF-Tu1 to both types of tRNA.

Interestingly, in EF-Tu proteins binding to canonical tRNA structures, an acidic residue is found at this position. Here, direct attractive electrostatic interactions with the phosphate backbone are not possible. Instead, these side chains were shown to form a single but important hydrogen bond with the amino group of a guanine residue in the 51 to 63 base pair of the T-stem (54). However, the presence of an acidic residue in *Bta* mt-EF-Tu is incompatible with an efficient interaction with armless tRNAs while introducing lysine at this position confers binding with a moderate apparent K_D_ of 96.8 nM (Fig. 6*A*). Taken together, it is conceivable that this region forms a direct interaction with the tRNA (*Bta* mt-EF-Tu: H-bond of aspartate with the T-stem of a canonical tRNA; *Rcu* mt-EF-Tu1 and probably also *Cel* mt-EF-Tu1: electrostatic attraction of lysine with the sugar-phosphate backbone of an armless tRNA).

Alternatively, these positions could contribute to a certain local structural environment in domain III that is involved in general tRNA binding. For example, in *E. coli* EF-Tu, E378 forms an H-bond to Y326 and a salt bridge to R318 (pdb entry 1OB2). In *Rcu* mt-EF-Tu1, the corresponding positions are replaced by Q322 and H330, possibly reflecting a co-evolutionary event to maintain the local structure in region F. Yet, the replacement of the acidic position D384 in *Bta* mt-EF-Tu (by lysine or alanine) improves the interaction with an armless tRNA substrate (Fig. 6*A*). This fact rather supports the scenario of a direct interaction with the armless tRNA. Here, crystal or cryo-EM structures of the ternary complex consisting of mt-EF-Tu1, GTP, and aminoacylated armless tRNA are needed to clarify this issue.

The possible tRNA interactions with acidic or basic side chains in domain III point to an evolutionary scenario for the adaptation to hairpin-like tRNAs. In contrast to canonical tRNAs, the presence of an acidic residue would be detrimental for armless tRNAs, as a hydrogen bond with a guanine residue is probably not possible since the elbow region is missing. Instead, the negative charges of the amino acid side chain and the tRNA backbone could result in an electrostatic repulsion, weakening the stability of the complex. Hence, nematode EF-Tu proteins recognizing non-canonical tRNAs have replaced this position with less impacting neutral side chains (S381 in *Tbr* mt-EF-Tu1) or a basic residue that even enhances tRNA binding by electrostatic interaction (K380 in *Rcu* mt-EF-Tu1 and K386 *Cel* mt-EF-Tu1). In *Tbr* mt-EF-Tu1, the situation seems to be unique, since the charge of position 381 has only minor effects on tRNA binding (Fig. 6*D*). Here, domain III may have evolved a different mode of interaction to accept canonical as well as non-canonical tRNA substrates.

**Figure 7:**
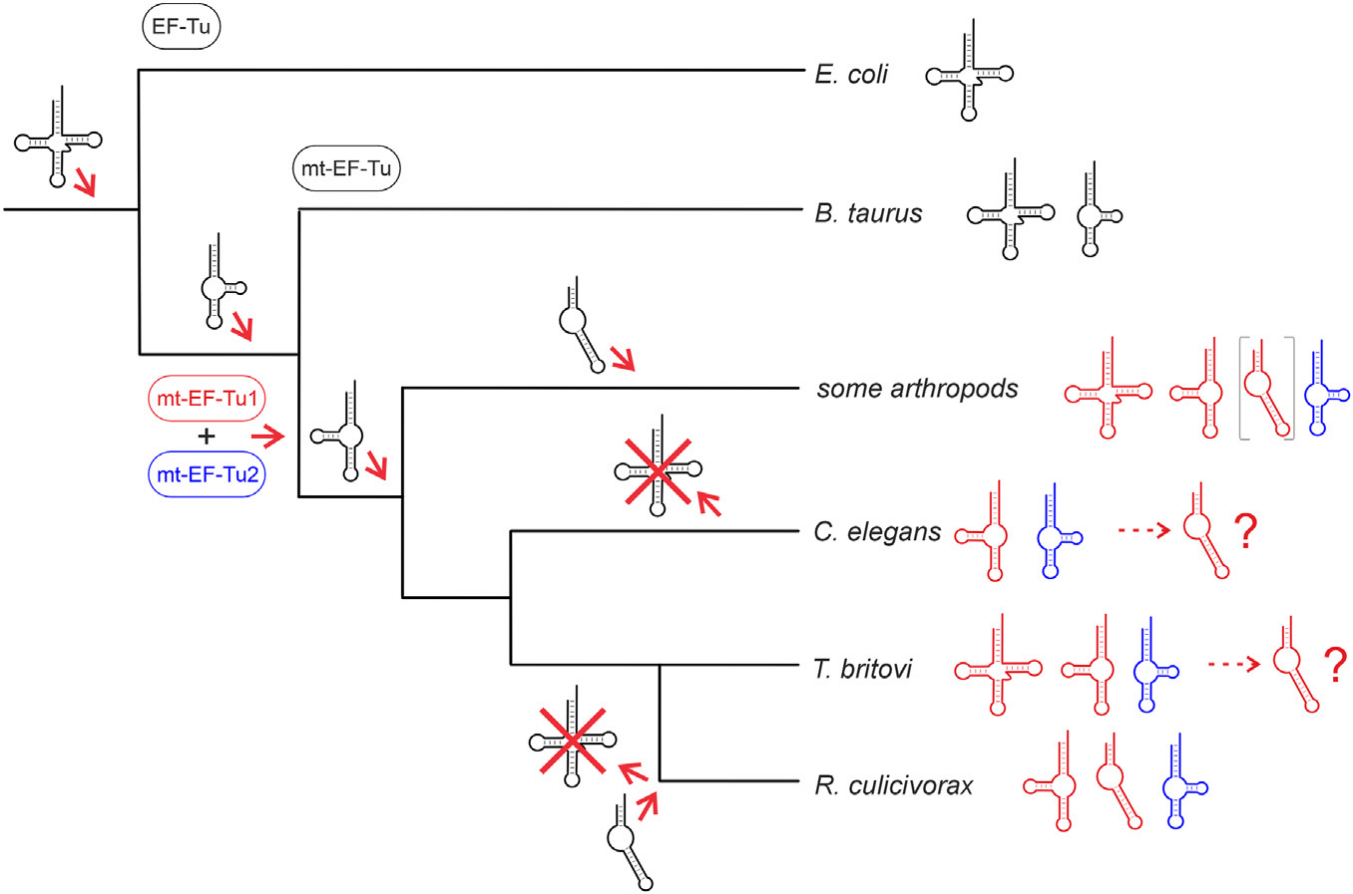
**Evolutionary scenario of mitochondrial tRNA truncations and EF-Tu adaptation.** The D-arm deletion in mt-tRNA^Ser^ (as it also occurred in mammals) still was compatible with the conventional mt-EF-Tu protein. In nematodes and some arthropods (insects and arachnids), a duplication of mt-EF-Tu occurred, resulting in mt-EF-Tu1 (*red*) and 2 (*blue*; tRNA substrates are colored according to the mt-EF-Tu specificities; in the case of arthropods, the armless tRNA is in *grey* brackets, as experimental evidence for mt-EF-Tu1 interaction is still lacking in these organisms). The latter one evolved as an elongation factor specific for mt-tRNA^Ser^ and represents the only case where the amino acid moiety of the charged tRNA is recognized. In arthropods and nematodes, some (in several cases all) mt-tRNAs were further truncated, resulting in T-armless and hairpin-like tRNAs. While in the arthropods as well as in *T. britovi*, some of the mt-tRNAs kept their cloverleaf structure, *C. elegans* and *R. culicivorax* only possess truncated transcripts lacking one (*C. elegans*) or even both arms (*R. culicivorax*). As mt-EF-Tu1 from *T. britovi* and *C. elegans* readily accept hairpin-like aa-tRNAs that are not present in their mitochondria, it is possible that this represents an evolutionary intermediate state, where such extremely truncated mt-tRNAs might evolve without detrimental effects (indicated by the question mark). However, a prerequisite for such a scenario is that also other tRNA-interacting proteins like synthetases, ribosome, *etc.* can deal with these transcripts.

With the discovery of hairpin-like tRNAs, and our findings on the concomitant EF-Tu adaptation, the evolutionary model concerning bizarre mitochondrial tRNAs and EF-Tu interaction can be extended (Fig. 7) (6). After appearance of the D-armless tRNA^Ser^, the gene for mt-EF-Tu was duplicated and diversified into the coding sequence of mt-EF-Tu1 and mt-EF-Tu2, and the latter then evolved to specifically recognize the D-armless tRNA (mainly by interaction with the serine moiety), while conventional tRNAs were bound by mt-EF-Tu1. In nematodes, many canonical mt-tRNAs further evolved into T-armless transcripts, requiring an adaptation of mt-EF-Tu1 that resulted in a considerable C-terminal extension. The positive charges in this element might have allowed a further truncation of mitochondrial tRNAs, resulting in hairpin-like, but functional structures. While these minimalistic tRNAs do not exist in *T. britovi*, it is possible that this situation represents an evolutionary intermediate and that future tRNA reductions might take place in *T. britovi* evolution, potentially independent of a basic position 381 in domain III. As *C. elegans* also carries a mitochondrial EF-Tu1 protein with both a C-terminal extension and a lysine residue in domain III (K386 in this case), it is also conceivable that this nematode might evolve hairpin-like tRNAs.

Taken together, the presented data show that besides the C-terminal extension, nematode mt-EF-Tu1 proteins carry an additional adaptation to interact with armless tRNAs in domain III, a structural element usually involved in the recognition of the elbow element of canonical tRNAs. A similar adaptation of a protein region to interact with structurally deviating tRNAs was described for the CCA-adding enzymes of *R. culicivorax* and *A. suum* (17, 20). In the *Rcu* enzyme, a β-turn of the catalytic core is known to position the tRNA 3′-end for nucleotide addition. To accept hairpin-like tRNA substrates, this element acquired several basic residues, allowing for an enhanced and efficient binding of the substrate. Furthermore, these and other nematode CCA-adding enzymes carry C-terminal extensions enriched in basic amino acid residues that contribute to tRNA binding (20). Obviously, the fact that certain protein regions adopt new functions to enable efficient interaction with non-canonical tRNAs represents a common strategy in the evolution of tRNA-binding proteins. It will be interesting to see whether other elements of the translational system in nematode mitochondria exhibit similar evolutionary adaptations. As a sum, it is surprising and highly fascinating that a process as central and highly conserved as protein biosynthesis exhibits such evolutionary plasticity. Currently, it is completely unclear whether such a tRNA minimalization has an evolutionary advantage or occurred purely coincidentally.

## Experimental procedures

### Cloning of recombinant EF-Tu proteins

The coding sequences of *R. culicivorax* mt-EF-Tu1 and mt-EF-Tu2 were identified and provided by Philipp Schiffer, University of Cologne (31). Open reading frames of *E. coli* EF-Tu (accession number AAN82549.1), *B. taurus* mt-EF-Tu (NP 776632.1), *C. elegans* mt-EF-Tu1 (NP 497623.1) and mt-EF-Tu2 (NP 491338.2) as well as *T. britovi* mt-EF-Tu1 (BAF32575.1) and mt-EF-Tu2 (BAF32576.1) were obtained from GenBank. Alignment and neighbor-joining phylogenetic tree reconstruction were performed using Clustal Omega and Jalview 2 (55, 56).

The open reading frame of *Eco* EF-Tu was PCR-amplified from *E. coli* total RNA and cloned into pET15b according to Arai *et al.* (57). All other EF-Tu ORFs were codon-optimized for expression in *E. coli*, synthesized, and cloned in pET-15b vector with an N-terminal His_6_-Tag (Azenta). All mt-EF-Tu sequences were cloned without the N-terminal mitochondrial target signal (Fig. 1*B*). Mutants and chimeric forms of EF-Tu open reading frames were generated using the QuikChange Site-Directed Mutagenesis Kit (Agilent).

### Expression and purification of recombinant proteins

*E. coli* BL21 (DE3) cells were transformed with plasmids encoding the different EF-Tu proteins and were grown in 400 ml of TB medium containing 100 μg/ml ampicillin at 30 °C (37 °C for *B. taurus* mt-EF-Tu). Protein expression was induced at an OD_600_ = 0.6 by adding IPTG to a final concentration of 0.1 mM (0.5 mM for *T. britovi* and *B. taurus* mt-EF-Tu). After 16 h of incubation at 16 °C (3 h at 37 °C for *B. taurus* mt-EF-Tu), cells were harvested and lysed by sonication in ice-cold buffer A (50 mM Tris/HCl pH 7.5, 60 mM NH_4_Cl, 7 mM MgCl_2_, 15% glycerol, 7 mM β*-*mercaptoethanol) with 15 μM GDP, 1 mg/ml lysozyme, 0.1 mM PMSF. The lysate was centrifuged for 30 min at 30,600 g, 4 °C, sterile-filtered and loaded onto a HisTrap FF column (Cytiva). Washing was performed with 15 column volumes of buffer A containing 5 μM GDP and 70 mM imidazole. Elution was carried out with five column volumes of buffer A with 5 μM GDP and 500 mM imidazole. Proteins were further purified by size-exclusion chromatography on a HiLoad 16/60 Superdex 75 pg column *(Cytiva) in* 50 mM Tris/HCl, pH 7.5, 150 mM NH_4_Cl, 7 mM MgCl_2_. Fractions containing the individual EF-Tu protein were combined and concentrated on a Vivaspin six column 10 kDa MWCO (Sartorius). Protein concentration was measured according to Bradford (58). Proteins were stored at −80 °C with 10% glycerol and an equimolar concentration of GDP.

### RNA production

tRNA and flexizyme transcripts bearing homogeneous 3′- and 5′-ends were generated by *in vitro* transcription as described (59). For radioactive labeling of tRNA, transcription was carried out in the presence of α-^32^P-ATP (3000 Ci/mmol). Due to ribozyme cleavage, the resulting tRNAs carried 5′-hydroxyl and 2′,3′-cyclophosphate ends. Since aminoacylation of tRNAs as well as their interaction with EF-Tu require 5′-phosphate and 2′,3′-hydroxyl ends, the transcripts were 5′-phosphorylated and 3′-dephosphorylated before use. To this end, 8 μM of RNA were incubated in 100 mM imidazole/HCl, pH 6.0, 100 μM ATP, 10 mM MgCl_2_, 10 mM β-mercaptoethanol, 20 μg/ml BSA, and 0.025 U/μl T4 PNK (New England Biolabs) for 2 h at 37 °C.

### tRNA aminoacylation

Aminoacylation of tRNA transcripts was carried out using the flexizyme system (32). 50 μM of tRNA were mixed with 50 μM of eFx in 50 mM HEPES/KOH, pH 7.5, 600 mM MgCl_2_, 50 mM Phe-CME and 20% DMSO for 2 h at 4 °C. For the analysis of the serine-specific *Rcu* mt-EF-Tu2, a modified flexizyme dFx and Ser-DBE were used to charge the individual tRNA substrates (6 h at 4 °C). Aminoacylated tRNA was ethanol-precipitated in the presence of 0.3 M potassium acetate, pH 5.2, and stored at −20 °C until use. The efficiency of aminoacylation was monitored by acidic gel electrophoresis on a 12.5% polyacrylamide gel. The percentage of the aminoacylated tRNA fraction was determined by densitometric analysis of the individual bands corresponding to charged and uncharged tRNA (32), resulting in an average charging efficiency of 73%. Accordingly, when calculating the binding curves, the obtained values were multiplied by the factor of 0.73.

### Ternary complex formation and DRaCALA assay

Prior to the formation of the ternary complex, EF-Tu:GDP was incubated for 30 min at 4 °C in buffer B (75 mM Tris/HCl, pH 7.5, 75 mM NH_4_Cl, 15 mM MgCl_2_, 2.5 mM phosphoenolpyruvate, 5 mU/μl pyruvate kinase, 1 mM DTT, 1 mg/ml BSA, 0.5 mM GTP). Consequently, GTP replaced the GDP in the complex, resulting in EF-Tu:GTP. Various concentrations of EF-Tu:GTP were then mixed with ^32^P-labeled aminoacyl-tRNA in buffer B to a final concentration of 4 nM tRNA.

The DRaCALA assay was carried out and evaluated as described (36). Briefly, 2 μl of the solution containing the ternary complex were spotted on nitrocellulose membrane (Cytiva, Freiburg, Germany), where protein (and bound aa-tRNA) is immediately immobilized, while unbound free tRNA is diffusing away from the spotting site. Bound and unbound tRNA was visualized by autoradiography, binding curves and dissociation constants of minimum three independent experiments were calculated by nonlinear regression using GraphPad Prism 7. For generation of binding curves, the complex formation was normalized by the percentage of aa-tRNA.

For wt EF-Tu proteins and key mutant versions, the active fraction of the preparations was measured using the DRaCALA assay. Increasing concentrations of EF-Tu protein were mixed with 750 nM of ^32^P-labeled aminoacylated tRNAs (in most cases mt-tRNA^Ile^; in the case of *Bta* mt-EF-Tu1 and *Eco* EF-Tu yeast tRNA^Phe^) to generate a titration curve. The active fraction was determined as described by measuring the amount of EF-Tu required to saturate the offered tRNA substrate (3). The percentage of active molecules (Table S1) was then used to calculate the dissociation constants from the input values of EF-Tu binding. For other protein variants (like the C-terminal deletions with almost identical affinity to the canonical tRNA), the active fractions of the corresponding wt proteins were used for these calculations, so that the obtained dissociation constants represent apparent K_D_ values, indicated by an asterisk. All obtained binding constants are summarized in Table S2 in the Supplementary Materials.

All experiments were done in at least three independent replicates.

## Data availability

Data will be available upon the reasonable request from the authors.

## Supporting information

This article contains supporting information (4, 5, 18, 24, 25, 60, 61, 62).

## Conflict of interest

The authors declare that they have no conflicts of interest with the contents of this article.
