# Supplementary material for: Armless hairpin-like tRNAs in Romanomermis culicivorax: Evolutionary adaptation of a mitochondrial elongation factor EF-Tu
Source: J Biol Chem. 2025 May 24;301(7):110294. doi: 10.1016/j.jbc.2025.110294 (PMC12221289; doi:10.1016/j.jbc.2025.110294)
Supplement: Supporting information [file mmc1.docx]

**Supporting Information**


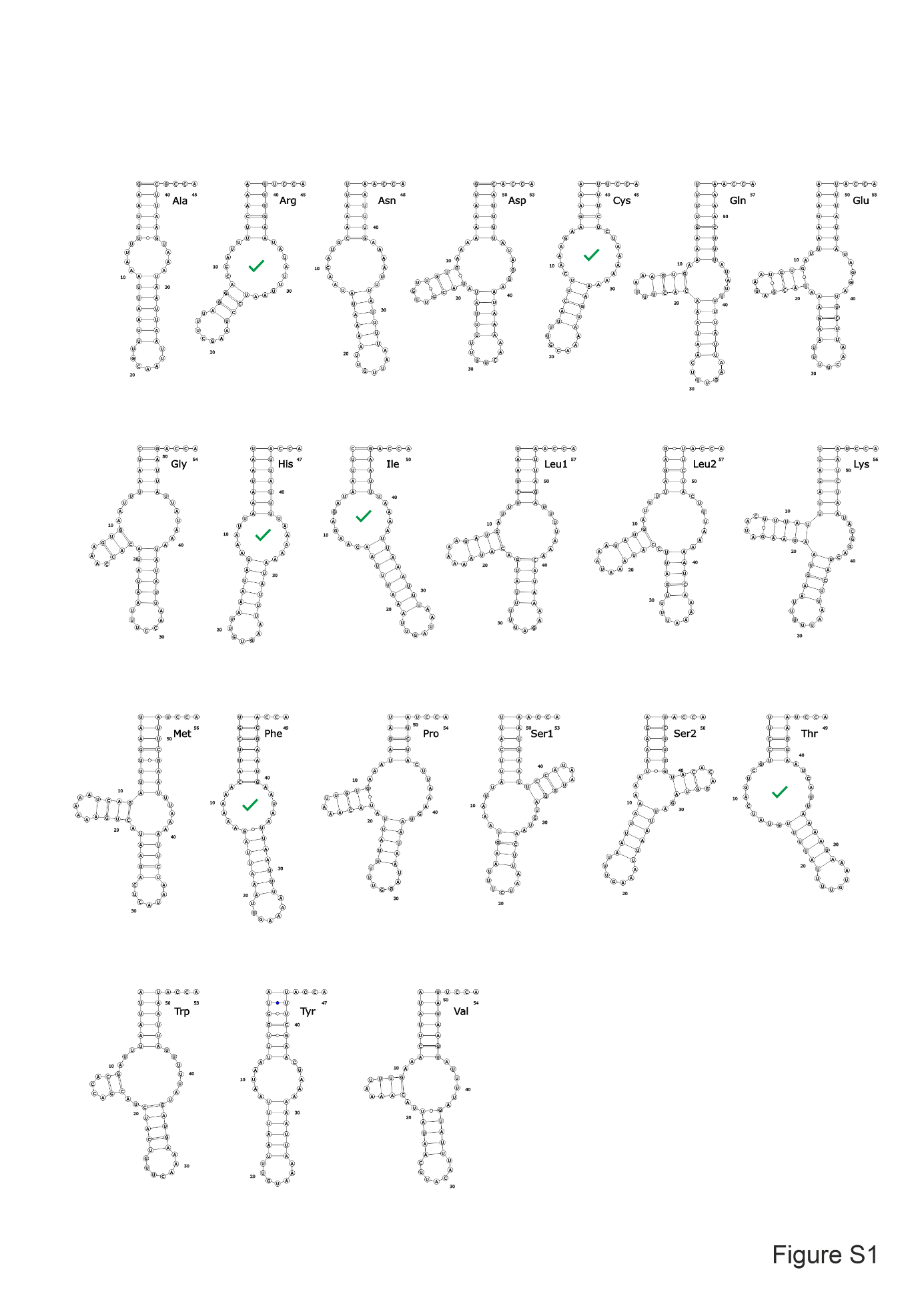


**Figure S1. Predicted mitochondrial tRNAs from *R. culicivorax*.**

The presented tRNA secondary structures are derived from Jühling et al: (1). Structure prediction was carried out using the Vienna RNA package (2) and fine-tuned by manual adjustments. Visualization was done using VARNA software (3). Green check marks indicate structures verified by sequence analysis of *in vivo* mt tRNAs and in-line probing of *in vitro* transcripts (4, 5).


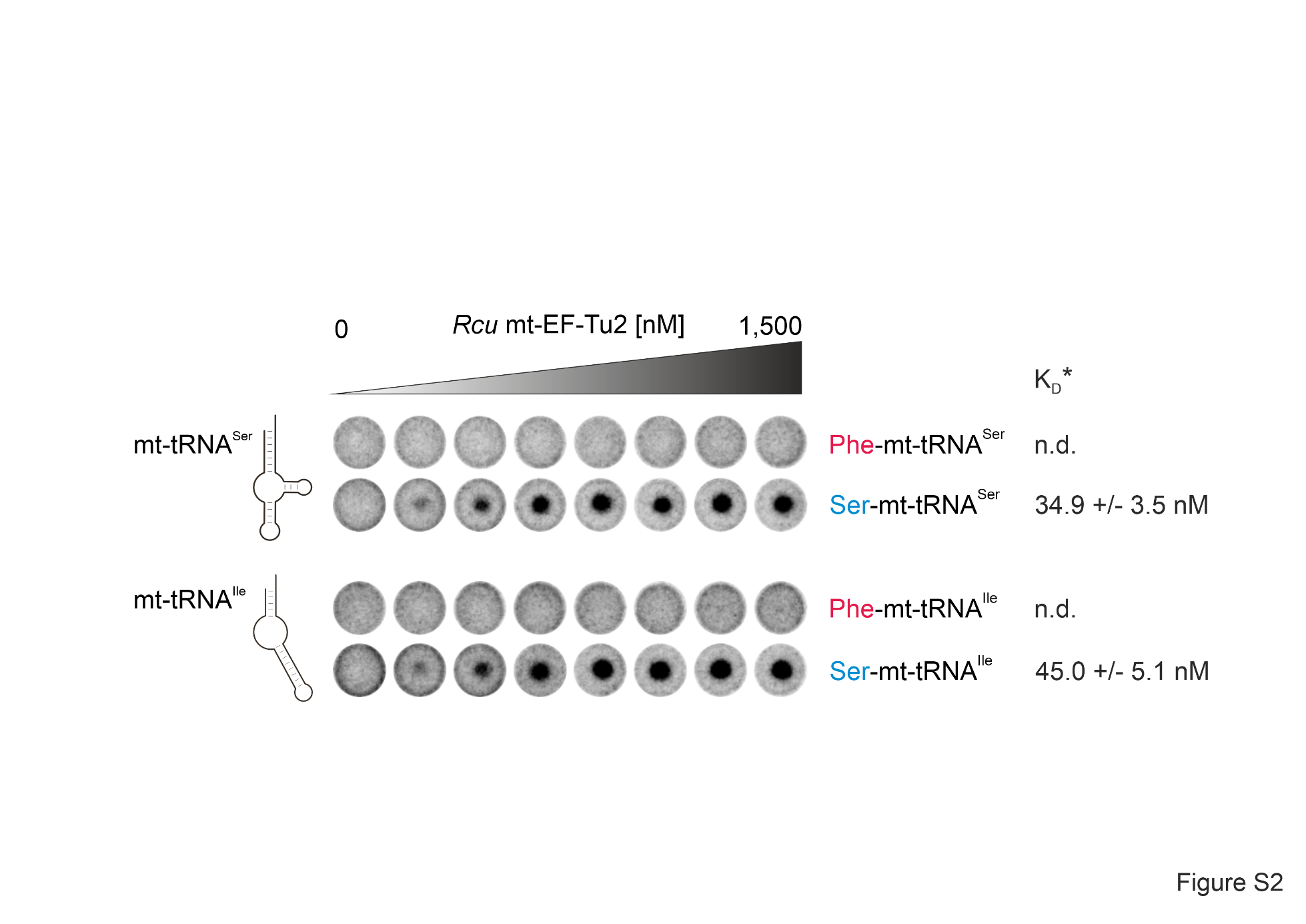


**Figure S2. Mitochondrial EF‑Tu2 from *R. culicivorax* is serine-specific.**

As its counterparts in *C. elegans* and *T. britovi*, this protein specifically recognizes the serine moiety (blue) (6, 7), regardless of whether the tRNA body is represented by the D-arm-lacking tRNA^Ser^ (upper panel) or the completely armless tRNA^Ile^ (lower panel) In contrast, the tRNAs charged with phenylalanine (red) are not recognized by *Rcu* mt‑EF‑Tu2. K_D_ values represent apparent values (K_D_*), as the active fraction of mt‑EF‑Tu2 was not determined.


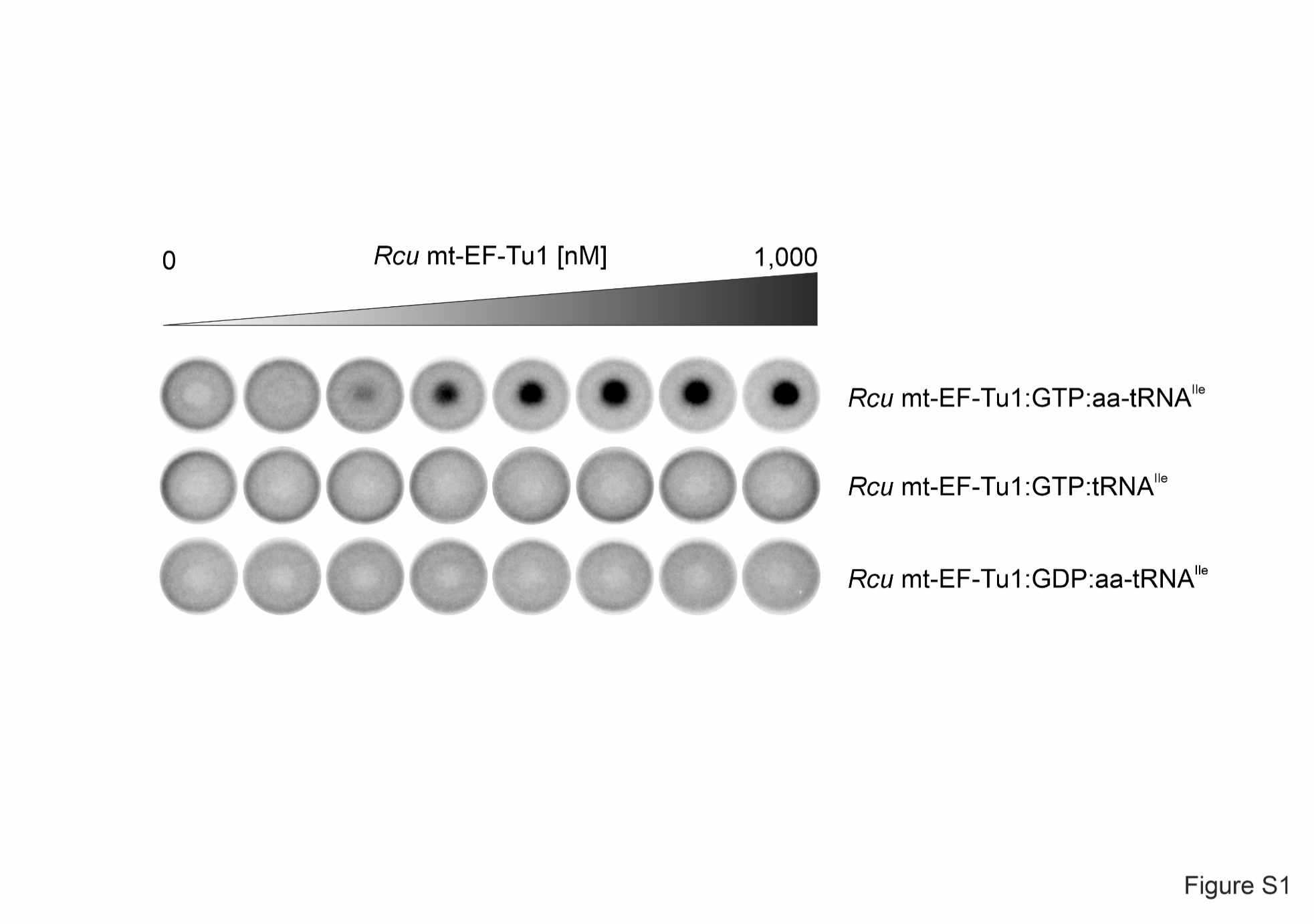


**Figure S3. A ternary complex is only formed with mt-EF-Tu1, GTP, and aminoacylated tRNA.**

As an example, the interaction of *Rcu* mt‑EF‑Tu1 with GTP and aminoacylated tRNA^Ile^ is shown in a DRaCALA experiment (upper row). Middle row: If the tRNA is not aminoacylated, no ternary complex is formed. Lower row: If GDP instead of GTP is offered, no ternary complex is formed either. These data indicate that mt‑EF‑Tu1 selectively interacts with aa-tRNA and GTP.

**
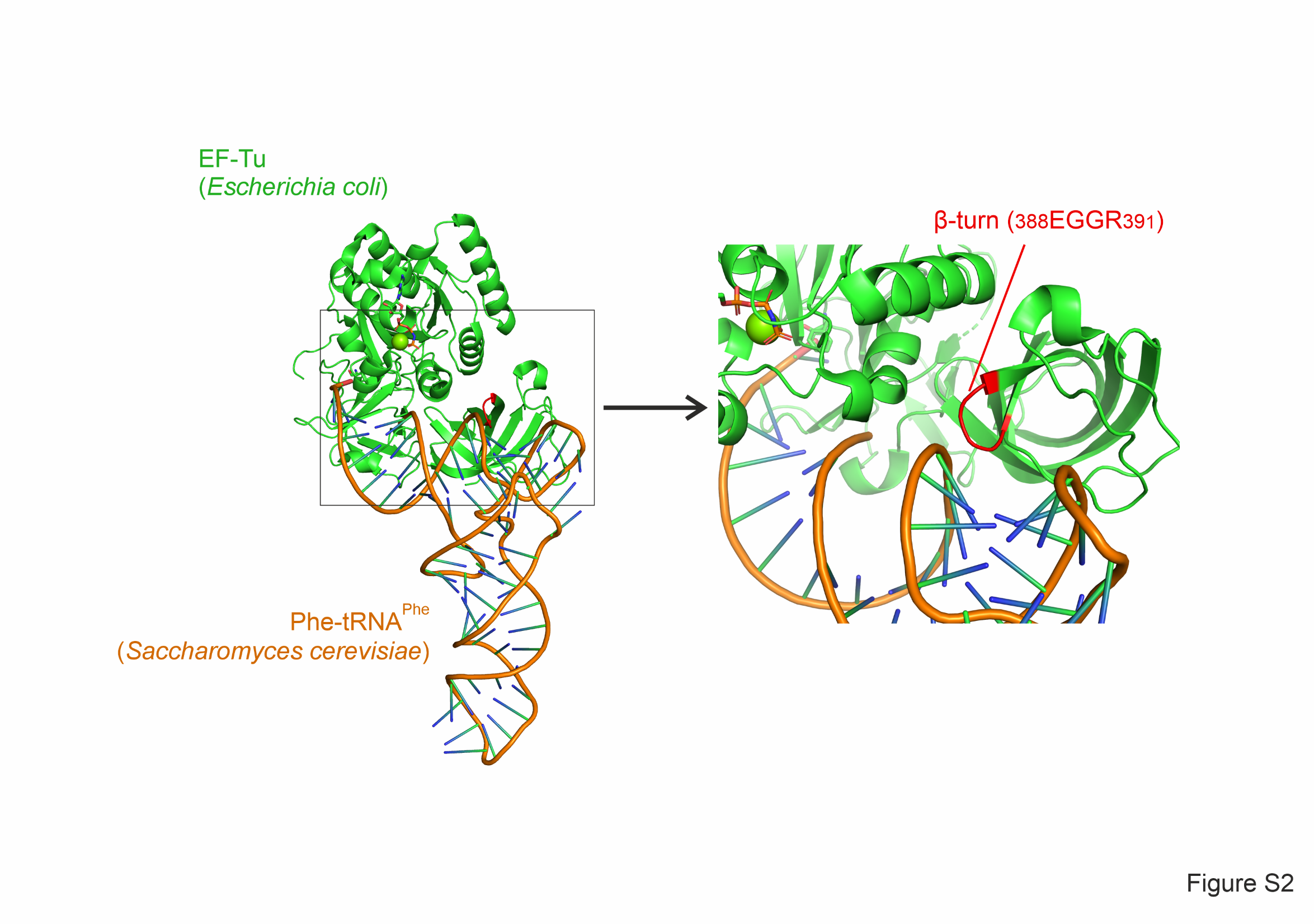
**

**Figure S4. Crystal structure of *Eco* EF‑Tu complexed with the GTP analog GDPNP and Phe-tRNA^Phe^ (pdb data base entry 1OB2).**

For reasons of clarity, the antibiotic kirromycin that is also bound in this complex is not shown. A beta-turn element (red) of EF‑Tu (green) is in close contact to the T‑arm of the bound tRNA (orange), representing a possible site of interaction with armless tRNAs in *Rcu* mt‑EF‑Tu1.

**
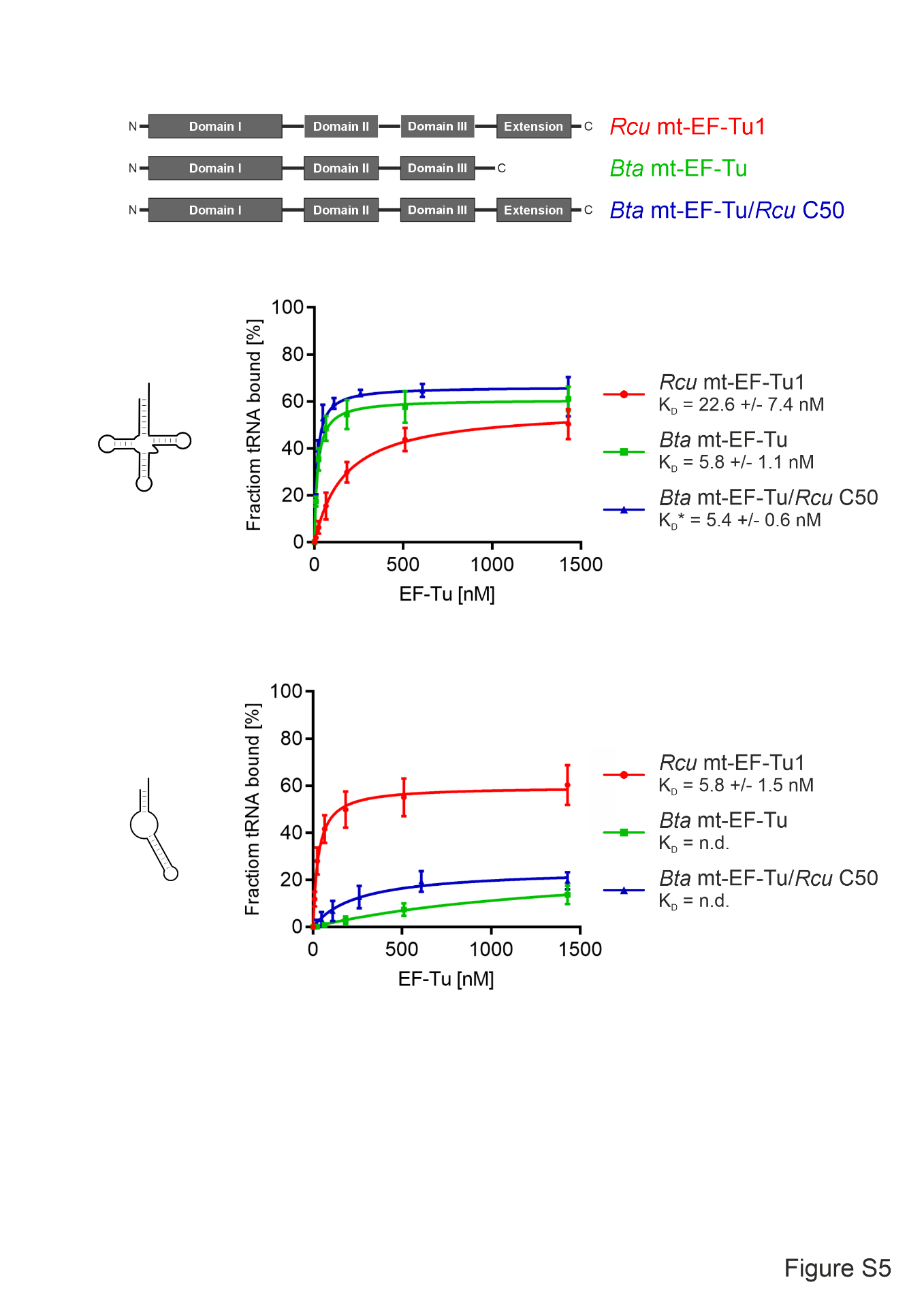
Figure S5. Binding behavior of *Bta* mt‑EF‑Tu carrying the C-terminal extension of *Rcu* mt‑EF‑Tu1 (*Bta* mt‑EF‑Tu/*Rcu* C50).**

**Upper diagram:** Like the wild type proteins *Bta* mt‑EF‑Tu and *Rcu* mt‑EF‑Tu1, the chimera efficiently recognizes a canonical tRNA substrate.

**Lower diagram:** In contrast, a noncanonical armless tRNA is only recognized by wt *Rcu* mt‑EF‑Tu1, but neither by wt *Bta* mt‑EF‑Tu nor by the chimeric bovine protein carrying the C-terminal extension of Rcu mt‑EF‑Tu1.

Error bars represent standard deviation (SD).

The asterisk indicates an apparent K_D_ value, where the active fraction of the wt protein was used in the calculation.

**Table S1. Active fractions of EF-Tu preparations**

Active fractions were determined in 2-3 independent experiments and showed highly consistent values in the same range as published for other EF-Tu proteins (8–10).

| **EF-Tu version** | **active fraction (%)** |
| --- | --- |
| ***Rcu* mt-EF-Tu1** |  |
| wt | 31 |
| ΔC50 | 29 |
| K380D | 23 |
| K380R | 53 |
| K380A | 30 |
| K380E | 25 |
| ***Bta* mt-EF-Tu** |  |
| wt | 33 |
| D384A | 33 |
| ***Eco* EF-Tu** |  |
| wt | 27 |
| ***Tbr* mt-EF-Tu1** |  |
| wt | 3 |
| S381K | 2 |
| S381D | 3 |
| ***Cel* mt-EF-Tu1** |  |
| wt | 8 |
| K386D | 9 |
|  |  |

**Table S2. K_D_ values of EF-Tu versions.**

Asterisks indicate apparent K_D_ values, where the corresponding wt active fraction was used

for calculation.

| **EF-Tu version** | **aa-tRNA** | | | |
| --- | --- | --- | --- | --- |
| ***Rcu* mt‑EF‑Tu1** | **canonical**  **(tRNA^Phe^)** | **armless**  **(mt-tRNA^Ile^)** | **T-armless**  **(mt-tRNA^Lys^)** | **D-armless**  **(mt-tRNA^Ser^)** |
| wt | 22.6 | 5.8 | 5.2 | 66.4 |
| ∆C3 | 23.6* | 8.8 | - |  |
| ∆C6 | 16.2* | 15.2* | - |  |
| ∆C9 | 27.2* | 26.1* | - |  |
| ∆C15 | 22.3* | 40.7* | - |  |
| ∆C18 | 20.8* | 50.4* | - |  |
| ∆C41 | 31.7* | 84.2* | - |  |
| ∆C50 | 26.1 | 82.1 | - |  |
| chimera region A | - | 17.1* | - |  |
| chimera region B | - | 18.6* | - |  |
| chimera region C | - | 4.8* | - |  |
| chimera region D | - | 14.6* | - |  |
| chimera region E | - | 10.3* | - |  |
| chimera region F | - | 221.7* | - |  |
| K380D | 197.4 | 70.4 | - |  |
| K380A | 58.4 | 33.4 | - |  |
| K380E | 150.6 | 149.5 | - |  |
| K380R | 60.5 | 16.5 | - |  |
| D381G | - | 4.9* | - |  |
| G382N | - | 7.8* | - |  |
| K383R | - | 11.3* | - |  |
| ∆C50/K380D | - | 349.9* | - |  |
| ***Cel* mt‑EF‑Tu1** |  |  |  |  |
| Wt | - | 17.1 | - |  |
| K386D | - | 62.6 | - |  |
| ***Tbr* mt‑EF‑T1** |  |  |  |  |
| Wt | - | 4.4 | - |  |
| S381K | - | 3.0 | - |  |
| S381D | - | 7.1 | - |  |
| ***Bta* mt‑EF-Tu** |  |  |  |  |
| Wt | 5.8 | n.d. | - |  |
| D384K | 76.6* | 96.8* | - |  |
| D384A | 11.5 | 60.1 | - |  |

References

1. Jühling, F., Pütz, J., Florentz, C., and Stadler, P. F. (2012) Armless mitochondrial tRNAs in Enoplea (Nematoda). *RNA biology* **9**, 1161–1166 10.4161/rna.21630 PMID 23018779

2. Lorenz, R., Bernhart, S. H., Höner Zu Siederdissen, C., Tafer, H., Flamm, C., Stadler, P. F., and Hofacker, I. L. (2011) ViennaRNA Package 2.0. *Algorithms for molecular biology : AMB* **6**, 26 10.1186/1748-7188-6-26 PMID 22115189

3. Darty, K., Denise, A., and Ponty, Y. (2009) VARNA: Interactive drawing and editing of the RNA secondary structure. *Bioinformatics* **25**, 1974–1975 10.1093/bioinformatics/btp250 PMID 19398448

4. Jühling, T., Duchardt-Ferner, E., Bonin, S., Wöhnert, J., Pütz, J., Florentz, C., Betat, H., Sauter, C., and Mörl, M. (2018) Small but large enough: structural properties of armless mitochondrial tRNAs from the nematode Romanomermis culicivorax. *Nucleic acids research* **46**, 9170–9180 10.1093/nar/gky593

5. Wende, S., Platzer, E. G., Jühling, F., Pütz, J., Florentz, C., Stadler, P. F., and Mörl, M. (2014) Biological evidence for the world's smallest tRNAs. *Biochimie* **100**, 151–158 10.1016/j.biochi.2013.07.034

6. Arita, M., Suematsu, T., Osanai, A., Inaba, T., Kamiya, H., Kita, K., Sisido, M., Watanabe, Y., and Ohtsuki, T. (2006) An evolutionary 'intermediate state' of mitochondrial translation systems found in Trichinella species of parasitic nematodes: co-evolution of tRNA and EF-Tu. *Nucleic acids research* **34**, 5291–5299 10.1093/nar/gkl526 PMID 17012285

7. Ohtsuki, T., Sato, A., Watanabe, Y., and Watanabe, K. (2002) A unique serine-specific elongation factor Tu found in nematode mitochondria. *Nat Struct Mol Biol* **9**, 669–673 10.1038/nsb826 PMID 12145639

8. Asahara, H., and Uhlenbeck, O. C. (2005) Predicting the binding affinities of misacylated tRNAs for Thermus thermophilus EF-Tu.GTP. *Biochemistry* **44**, 11254–11261 10.1021/bi050204y PMID 16101309

9. Pleiss, J. A., and Uhlenbeck, O. C. (2001) Identification of thermodynamically relevant interactions between EF-Tu and backbone elements of tRNA. *Journal of molecular biology* **308**, 895–905 10.1006/jmbi.2001.4612 PMID 11352580

10. Sanderson, L. E., and Uhlenbeck, O. C. (2007) Directed mutagenesis identifies amino acid residues involved in elongation factor Tu binding to yeast Phe-tRNAPhe. *Journal of molecular biology* **368**, 119–130 10.1016/j.jmb.2007.01.075 PMID 17328911
